# Supplementary figures and images for: Characterization of RNA interference in the cnidarian Nematostella vectensis reveals partial target silencing but lack of small RNA amplification
Source: PLoS Biol. 2026 Jan 5;24(1):e3003589. doi: 10.1371/journal.pbio.3003589 (PMC12768352; doi:10.1371/journal.pbio.3003589)

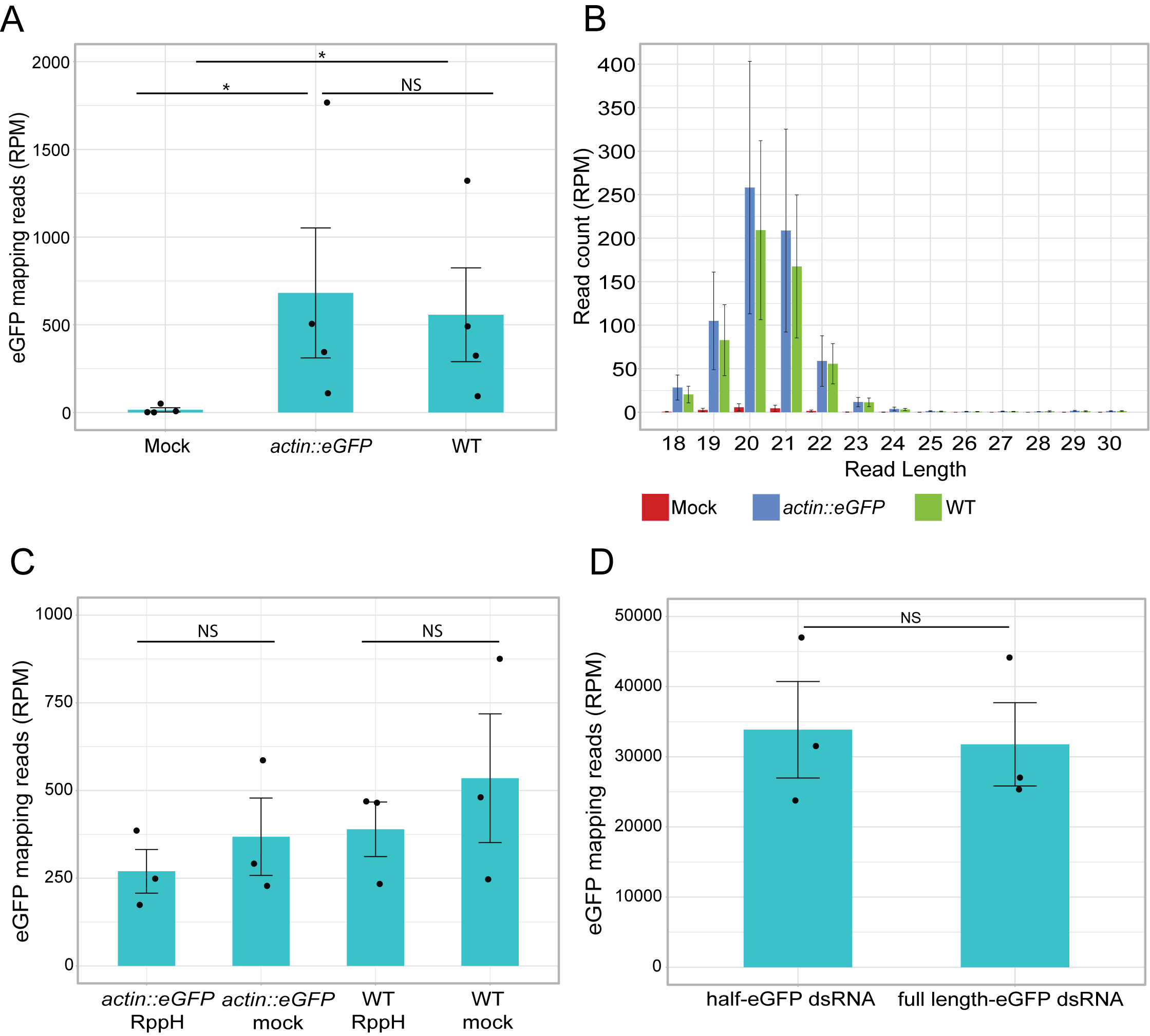

Supplement: S1 Fig — (A) Number of reads (RPM) mapping to eGFP sequence, from embryos injected with eGFP dsRNA (24 hpi). Actin::eGFP zygotes were injected with either dsRNA or mock injection mix. WT zygotes were injected with dsRNA. Statistical significance is shown for pairwise comparison (Kruskal–Wallis rank sum test, n = 4 biological replicates, p-value = 0.02, pairwise comparison with Wilcoxon rank sum exact test and FDR correction, p-values = 0.04, 0.04, 0.68). (B) Size distribution of reads mapping to eGFP sequence corresponding to the samples in (A). (C) Number of reads (RPM) mapping to eGFP sequence following treatment of RNA with RppH enzyme or mock treatment. The treatment was performed on RNA extracted from actin::eGFP or WT embryos injected with dsRNA carrying eGFP sequence (24 hpi). Statistical significance is shown for pairwise comparison (one-way ANOVA, n = 3 biological replicates, p-value = 0.49, pairwise comparison with Tukey’s HSD post-hoc test, p-values = 0.93, 0.81). (D) Number of reads (RPM) mapping to eGFP sequence, from embryos injected with either 3′ half- or full-length-dsRNA carrying eGFP sequence, or mock injection mix (24 hpi). Statistical significance is shown for pairwise comparison (two-tailed Student t test, n = 3 biological replicates, p-value = 0.82). Bar graphs show the mean; error bars represent the standard error. The data underlying this Figure can be found in S1 Data. (TIF) [file pbio.3003589.s001.tif]

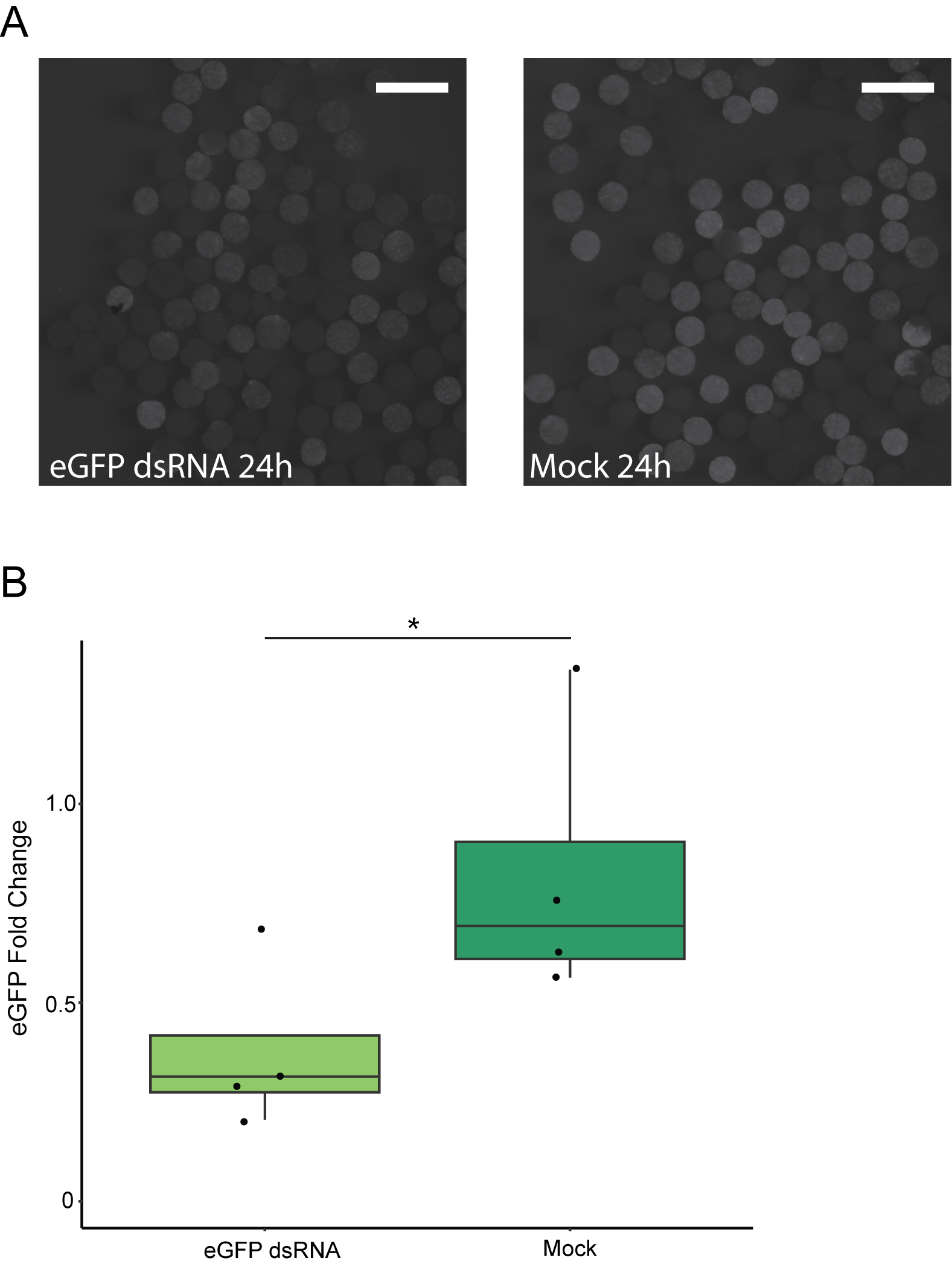

Supplement: S2 Fig — (A) Heterozygous actin::eGFP embryos showing dimmer eGFP fluorescence 24 hpi of dsRNA carrying eGFP sequence (left) compared to mock injection mix (right). Scale bars represent 500 µm. (B) eGFP transcript levels fold change measured by RT-qPCR, 24 hpi of dsRNA. Statistical significance is shown for pairwise comparison (one-tailed Student t test, n = 4 biological replicates, p-value = 0.02). Box plot indicates the median and interquartile range, with whiskers showing the minimum and maximum values. The data underlying this Figure can be found in S1 Data. (TIF) [file pbio.3003589.s002.tif]

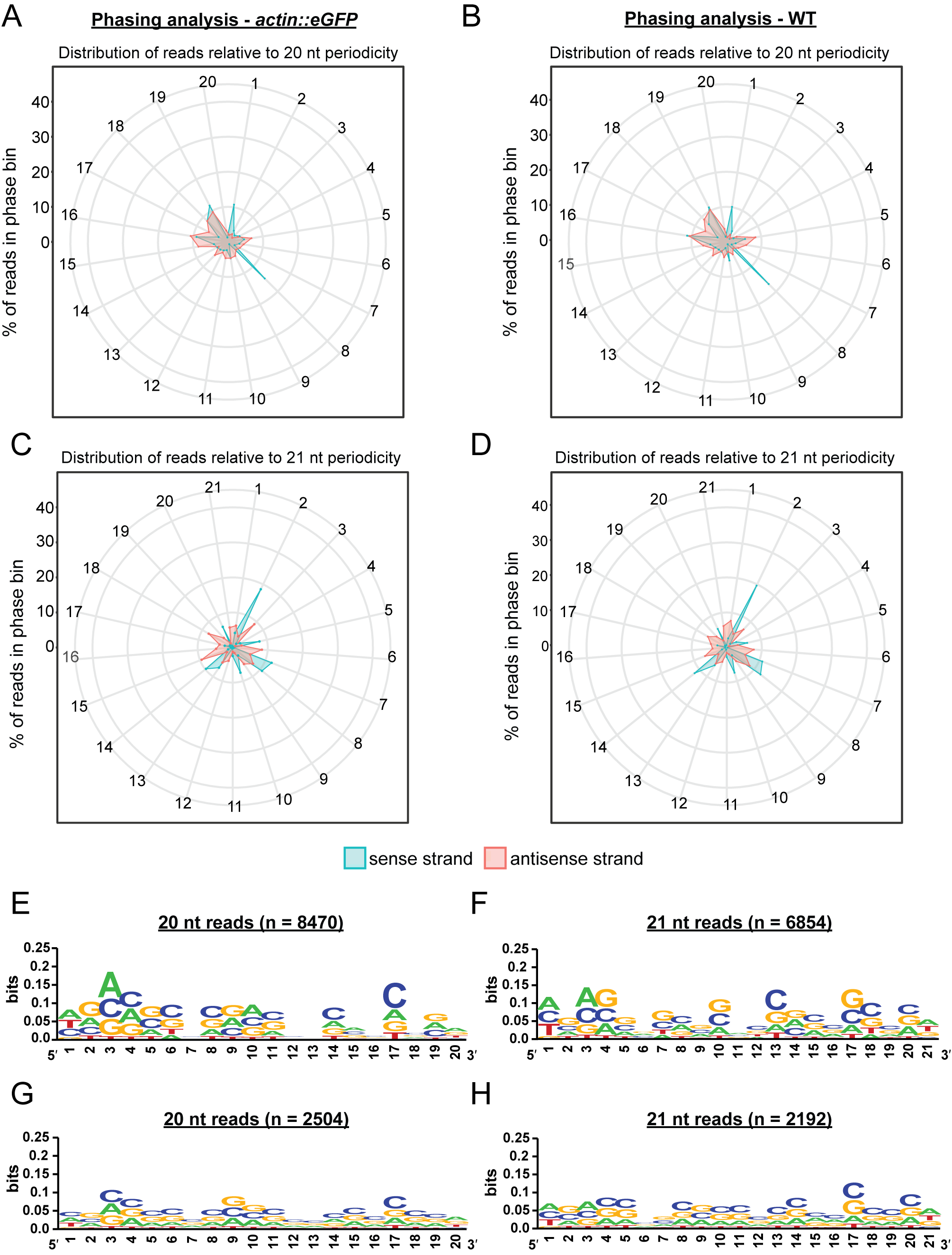

Supplement: S3 Fig — (A, B) Radar plot showing the average distribution of 20–22 nt siRNAs across 20 phasing registers along the entire eGFP sense and antisense strands. The radial distance represents the mean percentage of reads within each register, averaged from three biological replicates of dsRNA-injected actin::eGFP (A) or WT (B) zygotes. (C, D) Radar plot showing the average distribution of 20–22 nt siRNAs across 21 phasing registers along the entire eGFP sense and antisense strands. The radial distance represents the mean percentage of reads within each register, averaged from three biological replicates of dsRNA-injected actin::eGFP (C) or WT (D) zygotes. (E, F) Representation of sequence composition of total 20 (E) or 21 (F) nt eGFP-mapping reads, from three biological replicates of dsRNA-injected actin::eGFP zygotes. (G, H) Representation of sequence composition of unique 20 (G) or 21 (H) nt eGFP-mapping reads, from three biological replicates of dsRNA-injected actin::eGFP zygotes. Sequence logos were generated using WebLogo (78). The data underlying this Figure can be found in S1 Data. (TIF) [file pbio.3003589.s003.tif]

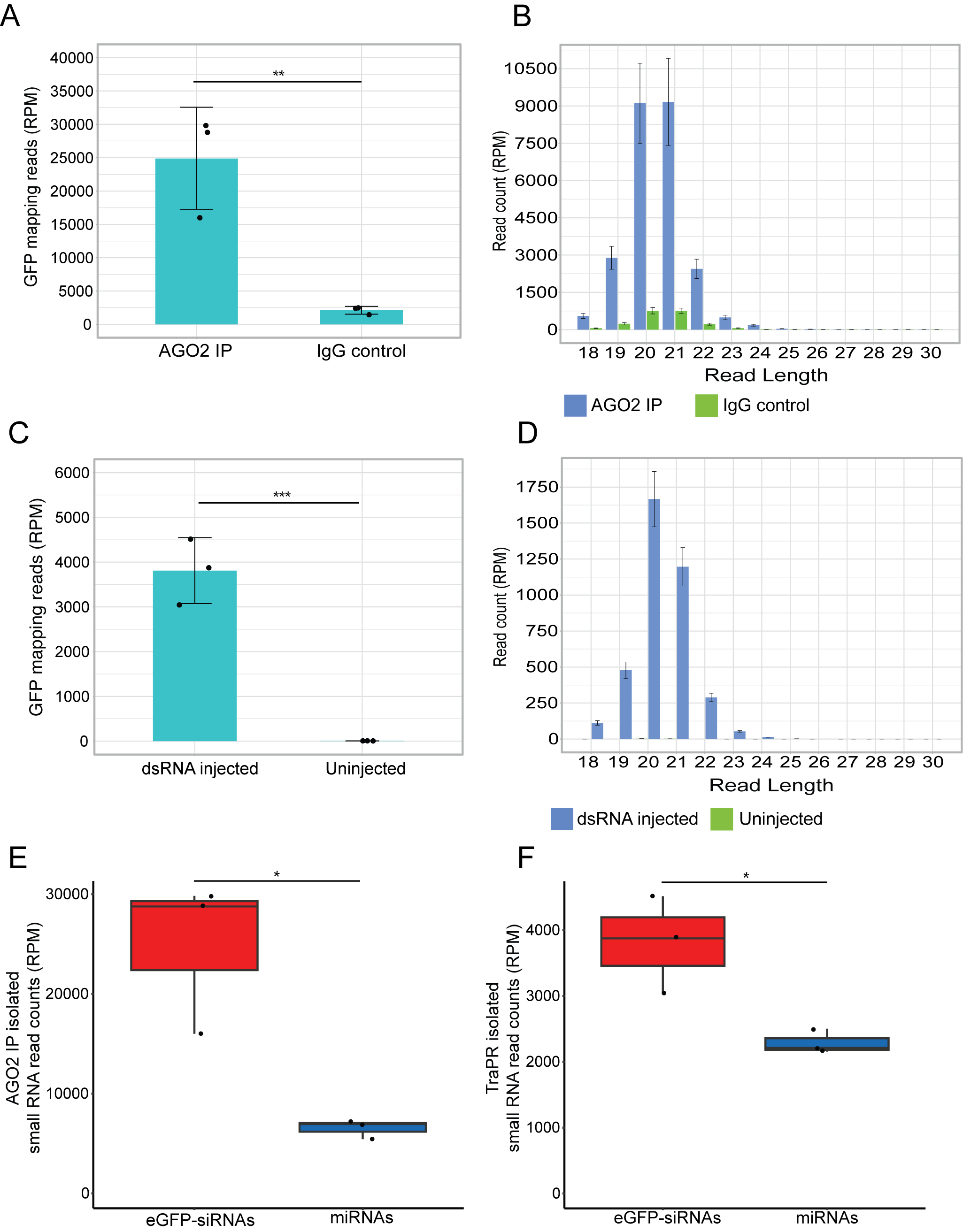

Supplement: S4 Fig — (A) Number of reads (RPM) mapping to eGFP sequence, recovered from AGO2 immunoprecipitated, or IgG antibody as negative control, samples of WT embryos injected with dsRNA carrying eGFP sequence (24 hpi). Statistical significance is shown for pairwise comparison (two-tailed Student t test, n = 3 biological replicates, p-value = 0.006). (B) Size distribution of reads mapping to eGFP sequence corresponding to the samples in (A). (C) Number of reads (RPM) mapping to eGFP sequence, recovered from RISC-isolated samples of WT embryos injected with dsRNA carrying eGFP sequence (24 hpi) or uninjected as negative control (24 hours old). Statistical significance is shown for pairwise comparison (two-tailed Student t test, n = 3 biological replicates, p-value < 0.001). (D) Size distribution of reads mapping to eGFP sequence corresponding to the samples in (C). (E) Number of reads (RPM) mapping to eGFP sequence compared to miRNA reads, recovered from AGO2 IP samples of WT embryos injected with dsRNA carrying eGFP sequence (24 hpi). Statistical significance is shown for pairwise comparison (two-tailed Student t test, n = 3 biological replicates, p-value = 0.01). (F) Number of reads (RPM) mapping to eGFP sequence compared to miRNA reads, recovered from RISC-isolated samples of WT embryos injected with dsRNA carrying eGFP sequence (24 hpi). Statistical significance is shown for pairwise comparison (two-tailed Student t test, n = 3 biological replicates, p-value = 0.02). Bar graphs show the mean; error bars represent the standard deviation. Box plots indicate the median and interquartile range, with whiskers showing the minimum and maximum values. The data underlying this Figure can be found in S1 Data. (TIF) [file pbio.3003589.s004.tif]

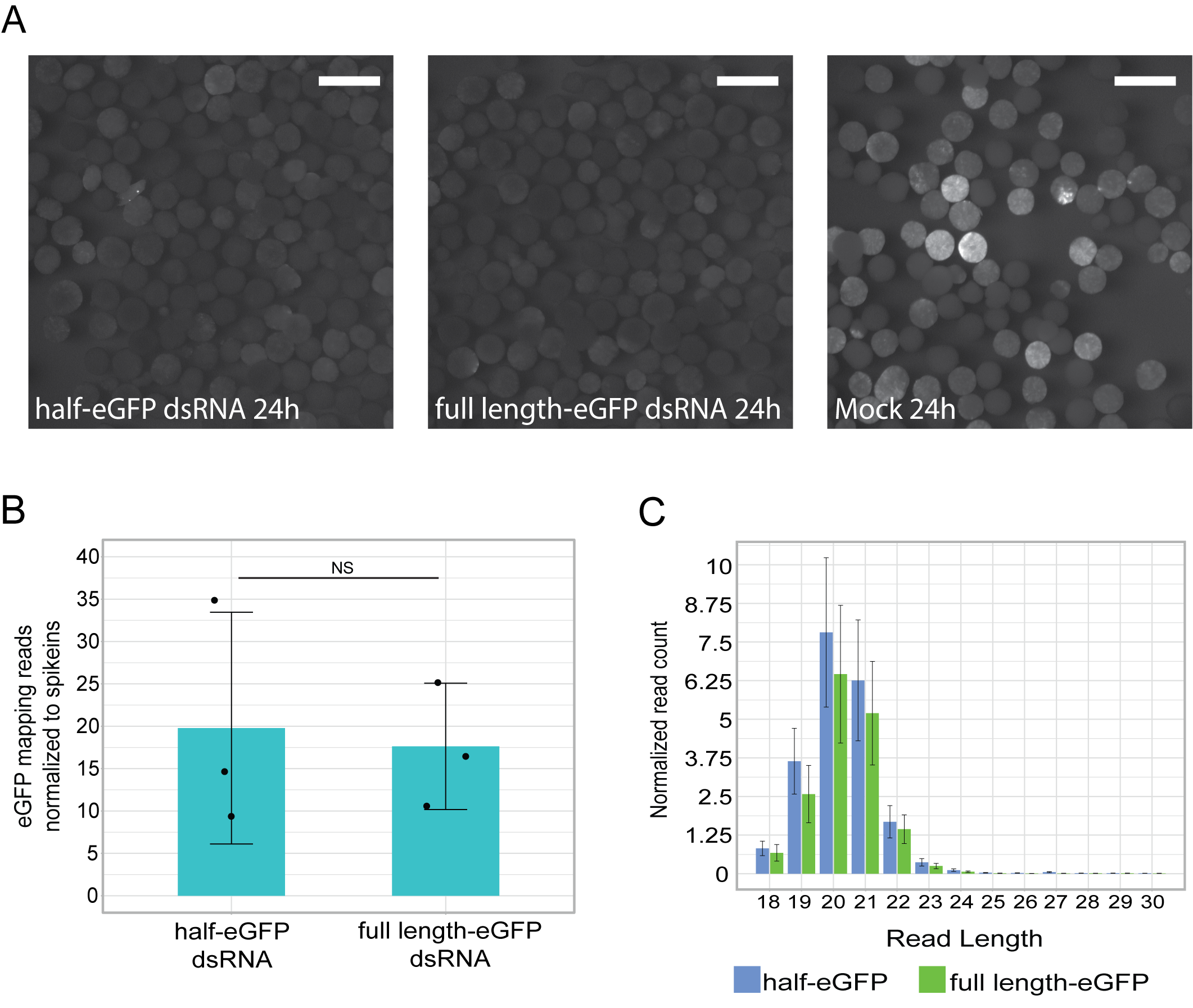

Supplement: S5 Fig — (A) Heterozygous actin::eGFP embryos showing dimmer eGFP fluorescence 24 hpi of 3′ half (left) or full length dsRNA carrying eGFP sequence (middle) compared to injection of mock injection mix (right). Scale bars represent 500 µm. (B) Normalized number of reads mapping to eGFP sequence, from embryos injected with either 3′ half or full dsRNA carrying eGFP sequence, or mock injection mix (24 hpi). Statistical significance is shown for pairwise comparison (two-tailed Student t test, n = 3 biological replicates, p-value = 0.82). (C) Size distribution of reads mapping to eGFP sequence corresponding to the samples in (B). Bar graphs show the mean; error bars represent the standard deviation in (B) and standard error in (C). The data underlying this Figure can be found in S1 Data. (TIF) [file pbio.3003589.s005.tif]

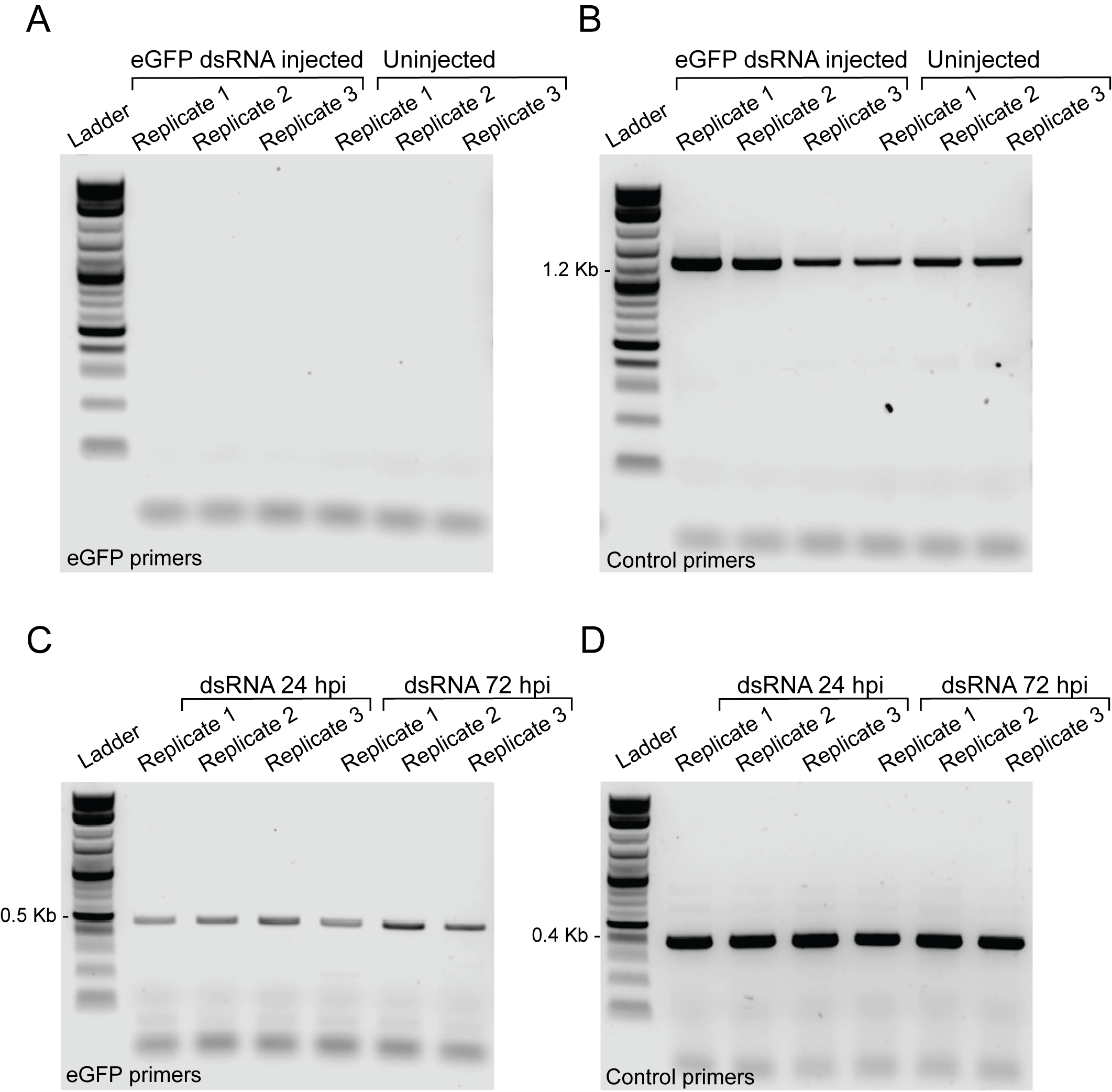

Supplement: S6 Fig — (A) No eGFP amplicons are seen when amplified from genomic DNA extracted at 72 hpi of WT animals with eGFP dsRNA. Uninjected WT animals serve as negative control (n = 3 biological replicates). DNA ladder is 1 kb Plus DNA Ladder in all panels. (B) Amplification of endogenous gene as positive control to (A) shows clear bands (n = 3 biological replicates). (C) Amplification of eGFP was successful when amplified from cDNA transcribed at 24 and 72 hpi of WT animals with eGFP dsRNA (n = 3 biological replicates). (D) Amplification of endogenous gene as positive control to (C) shows clear bands (n = 3 biological replicates). (TIF) [file pbio.3003589.s006.tif]

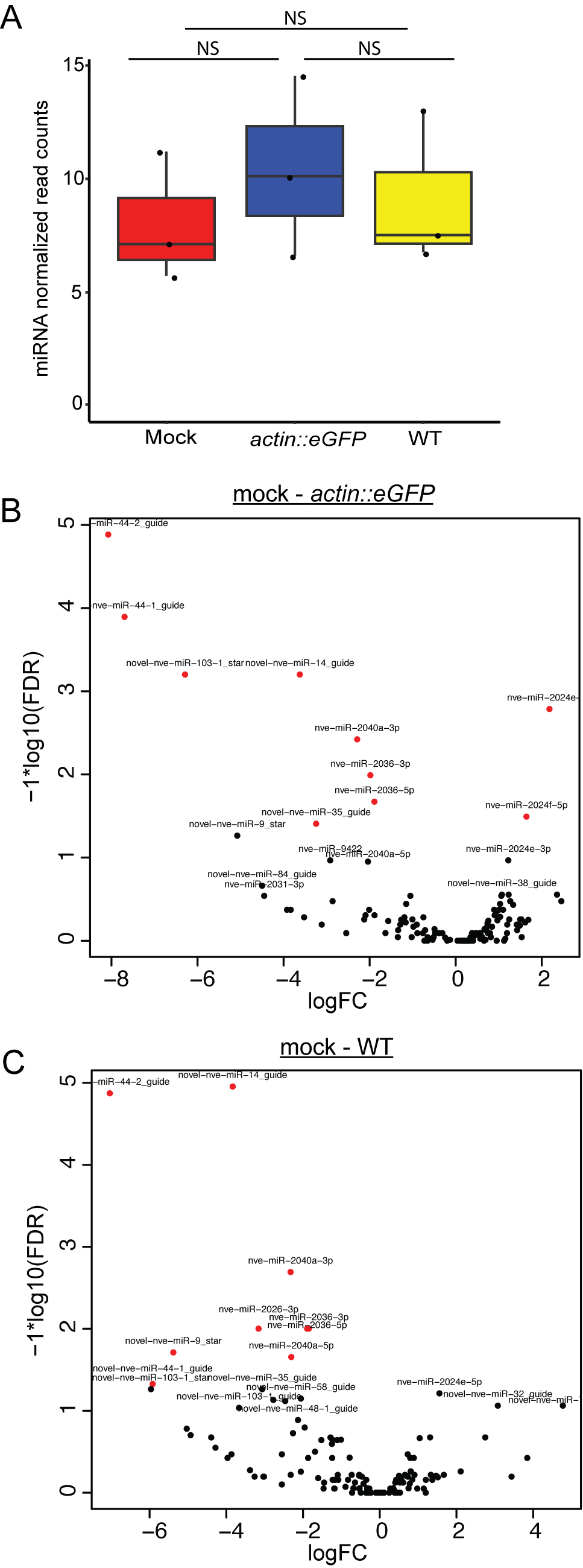

Supplement: S7 Fig — (A) Normalized total number of miRNAs reads from embryos injected with dsRNA (24 hpi). Actin::eGFP zygotes were injected with either dsRNA or mock injection mix. WT zygotes were injected with dsRNA. Statistical significance is shown for pairwise comparison (one-way ANOVA, p-value = 0.708, n = 3 biological replicates, pairwise comparisons with Tukey’s HSD post-hoc test, p-values = 0.68, 0.91, 0.89). (B, C) Differentially expressed miRNAs in actin::eGFP (B) and WT (C) embryos following injection of dsRNA (24 hpi). Box plot indicates the median and interquartile range, with whiskers showing the minimum and maximum values. The data underlying this Figure can be found in S1 Data. (TIF) [file pbio.3003589.s007.tif]

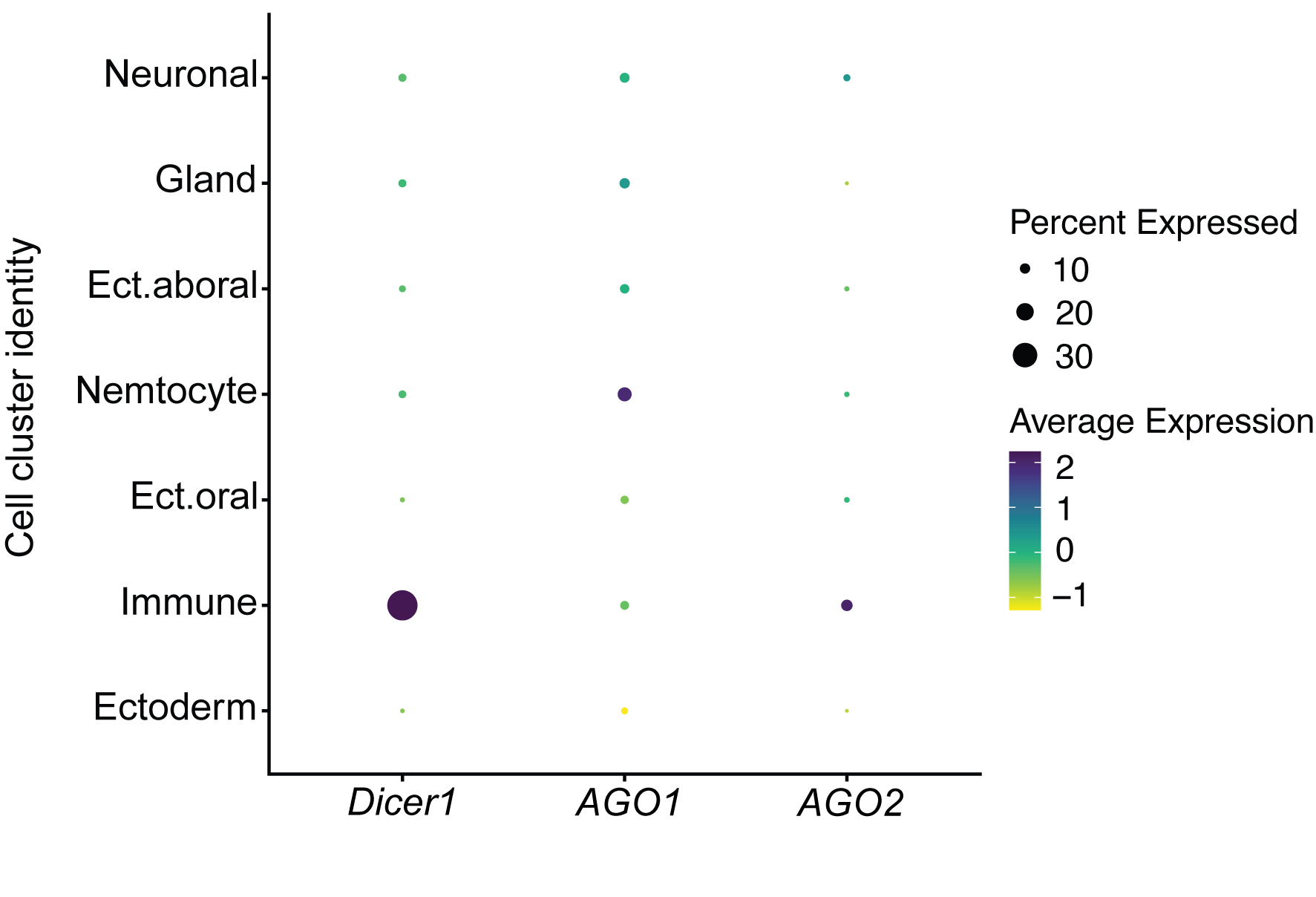

Supplement: S8 Fig — Dot plot showing the percentage of cells expressing each gene (dot size) and the mean normalized expression level (color intensity) across annotated cell clusters from single-cell RNA-seq data. (TIF) [file pbio.3003589.s008.tif]

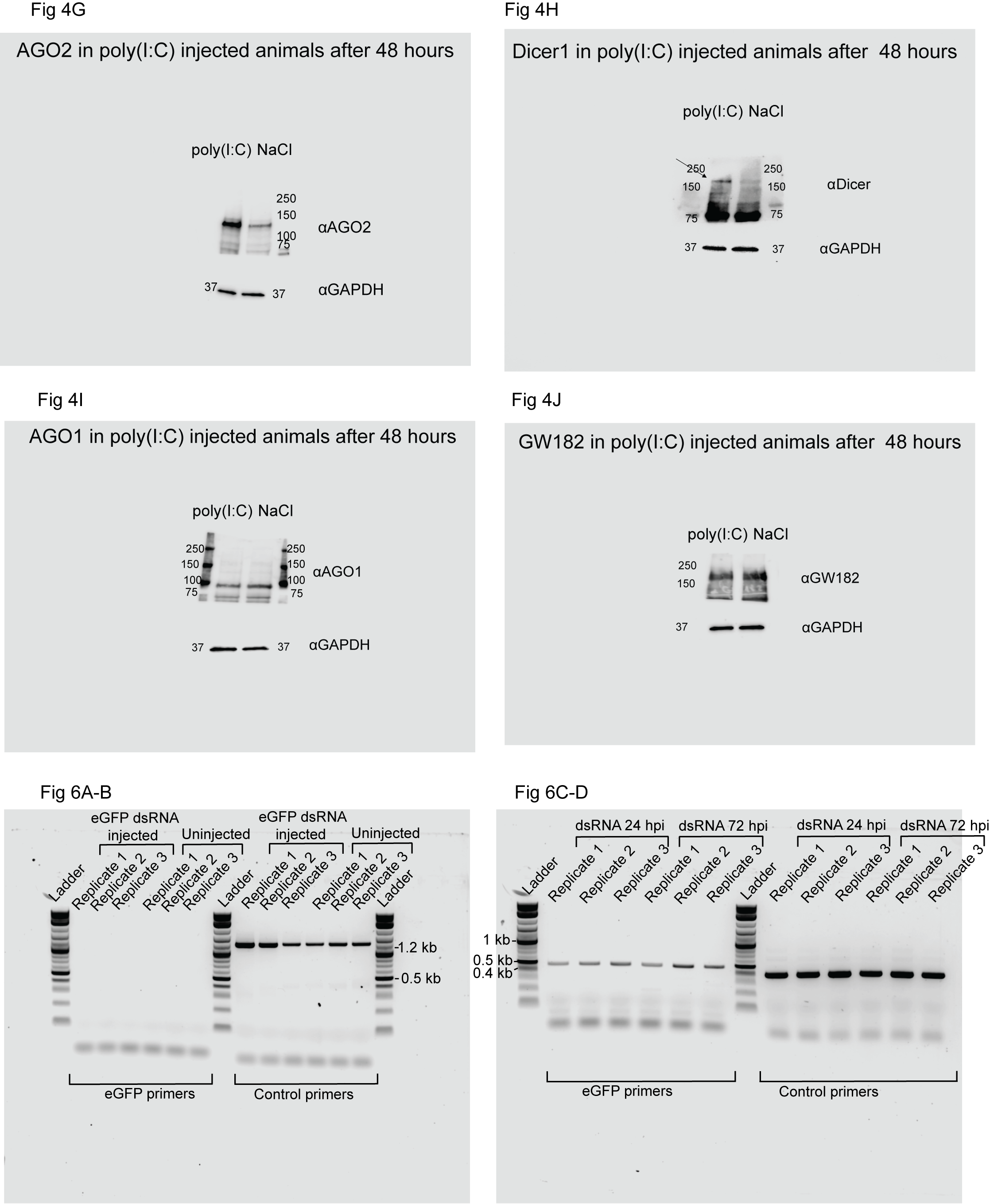

Supplement: S1 Raw Images — (TIF) [file pbio.3003589.s009.tif]
