# Supplementary material for: Characterization of RNA interference in the cnidarian Nematostella vectensis reveals partial target silencing but lack of small RNA amplification
Source: PLoS Biol. 2026 Jan 5;24(1):e3003589. doi: 10.1371/journal.pbio.3003589 (PMC12768352; doi:10.1371/journal.pbio.3003589)
Supplement: Attachment — Submitted filename: Response to reviewers.pdf [file pbio.3003589.s012.pdf]

Dear Dr. Hodge,

We thank you and the referees for the helpful and constructive comments. We were pleased to learn that the three referees found our paper of interest. Please see below our point-by-point response to all comments.

Kind regards,

Yael Admoni and Yehu Moran on behalf of all authors

Reviewer remarks:

Reviewer #1: This work interrogates the activity of dsRNA-driven RNAi in the model organism Nematostella vectensis. The topic is of great interest, as it touches the evolution of immune responses in eukaryotes. The experiments are well-designed, conclusions are clear and the manuscript is very well-written. I have a few suggestions that will help reinforce the authors' claims, but they are fairly minor. Provided answers, I recommend this work for publication. Thank you for the interesting read!

1. Fig 1A-B, could the authors quantify the fluorescence intensity on the images? It would nicely complement the RT-qPCR data.

Answer:

We thank the reviewer for this helpful comment. We have added a quantification of fluorescence intensity to the manuscript (Figure 1E and Supplementary file 2).

2. Fig 1, the authors should repeat this experiment in the context of Dicer1 and Ago2 KD, to formerly demonstrate that GFP silencing depends on a canonical dsRNA-driven RNAi.

Answer:

We thank the reviewer for this comment. We agree that such experiment could have supported that eGFP silencing depends on a dsRNA-driven RNAi. Unfortunately, since AGO2 and Dicer1 in *Nematostella* also take part in the miRNA system, knocking them out leads to severe developmental defects (Modepalli et al., 2018, DOI: 10.1371/journal.pgen.1007590; Fridrich et al., 2020, DOI: 10.1038/s41467-020-20003-8). In addition, AGO2 and Dicer1 are maternally deposited as proteins, which would make knockdown inefficient at the first hours post injection (hpi) and given that the immune response peaks at 24 hpi, would not fit our assays. We have added two experiments that validate the loading of eGFP-derived siRNAs into AGO2 (Supplementary Figure 4), by performing AGO2 immunoprecipitation and Trans-

kingdom rapid affordable Purification of RISCs (TraPR). We believe that these additional data confirm that the silencing of eGFP depends on the siRNA pathway.

3. The authors demonstrated in a previous work that poly(I:C) stimulation translates into an "inflammatory" response that bears similarities with the interferon response of mammals (ref 26). Does this also happen when dsRNA-GFP is transfected, as mentioned in line 276? I am wondering if the modest and transient silencing effect observed in Fig 1 could be due to a concomitant effect of inflammation, that could antagonize dsRNA-driven RNAi, as demonstrated in mammalian differentiated cells. Or, as the authors point out, the two responses could synergize. It is outside the scope of this work, but it would be interesting to repeat the experiment in Fig 1 with a KD of NvRLRs.

Answer:

The transcriptional response of dsRNA receptor NveRLRb and additional interferon-related genes to injection of eGFP-dsRNA has been tested in the manuscript mentioned by the reviewer (Lewandowska et al., 2021, DOI: 10.1093/molbev/msab197), where it was shown to be similar to the response to poly(I:C). As mentioned by the reviewer, in this paper we discuss the possibility that the interferon-like response and RNAi are synergized in *Nematostella*, and we also discuss the possibility that activation of apoptosis, which is seen upon poly(I:C) injection (Kozlovski et al., 2024, DO: 10.1371/journal.ppat.1012320), could explain the moderate RNAi effect. We agree with the reviewer that repeating the experiments presented in this paper with a knockdown of NveRLRs would be interesting, although beyond the scope of this paper. Notably, just like Dicer1 and AGO2, the two NveRLRs are maternally deposited, making the knockdown approach quite inefficient.

4. The production of siRNAs derived from dsRNA is quite compelling. I suggest that the authors complete the analysis by looking at the phasing of siRNAs as well as the 5' biais, two measures that are usually included in siRNA analysis in species such as fruit flies or C. elegans.

Answer:

We thank the reviewer for this valuable suggestion. We included a phasing analysis, which shows that siRNAs are not strongly phased, indicating that Dicer processes dsRNA in a different manner than what was shown in mammalian cells infected by a single-stranded RNA virus (Maillard et al., 2013, DOI: 10.1126/science.1241930) (Supplementary Figure 3A-D). The weak phasing resembles Dicer products in mouse cells that were transfected with RNA hairpin with long single-strand overhangs (Demeter et al., 2019, DOI: 10.26508/lsa.201800289), which means that in our experiments,

despite the dsRNA being accessible from both ends, it was processed by cleavage at internal sites rather than slowly progressing from one end. The dispersed siRNA mapping pattern agrees with this finding (Figure 2C and D).

We have also added a representation of the sequence composition of siRNA reads, which revealed no sequence bias, including in the 5′ (Supplementary Figure 3E-H). Notably, the 5′ nucleotide bias in *D. melanogaster* siRNAs is also minimal in some cases (Ghildiyal et al., 2010, DOI: 10.1261/rna.1972910).

5. Fig 3 shows that there is no secondary RNA generated via RdRp activity on target mRNA in this system. The authors also mention the case of insects, in which viral RNA is reverse transcribed into vDNA and then transcribed to generate a pool of siRNAs. The authors' experimental setup does not allow to assess if a similar mechanism may be at play in Nematostella. To test this easily, the authors could inject dsRNA coding for a sequence of their choice, and then perform PCR to assess if the dsRNA was reverse transcribed.

Answer:

We thank the reviewer for this helpful comment. As suggested, we injected eGFP dsRNA to WT zygotes at three biological replicates and extracted genomic DNA after 72 hours from the time of injection. We conducted PCR using eGFP-specific primers and primers for an endogenous gene as a positive control. The results of this experiment show no amplification of eGFP from injected animals, indicating that the dsRNA was not reverse transcribed into viral DNA (Supplementary Figure 6A and B).

6. Injection of dsRNA is performed at the zygote stage, and silencing is analyzed 24h later. Could the authors discuss how dsRNA-driven RNAi and RLR-driven inflammation could behave in the adult? Do they expect the two responses to be active in the same cells, or could there be some sort of functional specialization (different responses in different cell types), as seen with antiviral RNAi in mammalian oocytes and stem cells?

Answer:

We have added to the Discussion our suggestion regarding how dsRNA-driven RNAi could behave in adults. As discussed in Kozlovski et al., 2025 (DOI: https://doi.org/10.1101/2025.01.24.63469, preprint), an overlap of differentially expressed genes was observed between poly(I:C) triggered *Nematostella* embryos (Lewandowska et al., 2021, DOI: 10.1093/molbev/msab197) and 2'3'-Cyclic GMP-AMP (cGAMP) triggered 4-weeks-old juvenile polyps (Margolis et al., 2012, DOI: 10.1073/pnas.2109022118). Moreover, similar genes were found to be expressed in immune cell clusters between *Nematostella* embryos and adults (Cole et al., 2024, DOI:

10.1186/s12983-024-00529-z). Therefore, we suggest that dsRNA-driven RNAi likely behaves in adults similarly to embryos.

Single-cell data obtained by Kozlovski et al. (2025) reveal an immune cell cluster, indicating that a specialized cell population in *Nematostella* performs antiviral activity. *NveDicer1* and *NveAGO2* are enriched in these immune cells; hence, antiviral RNAi is likely a function provided by these cells (Supplementary Figure 8).

7. The authors should not use "antiviral RNAi" for the mechanism they are studying. There is no virus involved in these experiments. dsRNA-driven RNAi is more appropriate.

Answer:

We thank the reviewer for raising this important point. We would like to address this comment together with those raised by reviewers #2 and #3. First, we would like to explain our system's limitations. *Nematostella* currently lacks culturable natural viruses or isolated viruses that infect it under lab conditions. Moreover, *Nematostella* does not have an available cell culture, making direct viral infection experiments very challenging. In previous attempts, we tested several viruses capable of infecting both vertebrate and invertebrate hosts (e.g., Sindbis virus, West Nile virus) but observed no successful infection. Notably, the *C. elegans* community faced similar challenges for many years as their model organism lacked any culturable viruses until the Orsay virus was found (actually in an RNAi-deficient natural population!).

To overcome these limitations, we simulated viral infection by (i) injection of long dsRNA, a canonical replication intermediate of RNA viruses, and (ii) injection of Flock House virus (FHV) RNA1 mRNA encoding the viral RdRp, an approach that was used by *C. elegans* antiviral immunity researchers in the past. Our earlier work demonstrated that injection of dsRNA (a similar molecule was used in this study) or poly(I:C) into *Nematostella* zygotes triggers upregulation of immune-related genes, including components of the siRNA pathway (AGO, Dicer and RdRps), as well as homologs of the interferon pathway genes such as RLRs, Interferon regulatory factors (*IRF*s), RNase L, guanylatebinding proteins (*GBP*s), 2′-5′-oligoadenylate synthetase 1(*OAS1*) and nuclear factor kappa-light-chain-enhancer of activated B cells (*NF-κB*) (Lewandowska et al., 2021, DOI: 10.1093/molbev/msab197). This upregulation indicates that dsRNA injection activates an antiviral immune response that includes siRNA pathway components.

In this current study, we show that FHV-eGFP mRNA injection leads to strong upregulation of *Dicer1* and *AGO2*, accompanied by a mild reduction in eGFP fluorescence, paralleling the effects observed with direct dsRNA injection (Figure 4A-F). Since *Dicer1* and *AGO2* are not upregulated in response to control mRNA, this pattern

supports that the self-replicating FHV RdRp mRNA effectively simulates viral replication and triggers the antiviral siRNA pathway.

Additionally, siRNA-specific components are upregulated while miRNA-specific components (AGO1, GW182) are mildly downregulated upon poly(I:C) exposure (Figure 4G-N), reinforcing that this response specifically involves the siRNA machinery as an immune mechanism. Consistent with this interpretation, single-cell RNA-seq data from an *RLRb::mCherry* reporter line stimulated with poly(I:C), identified a distinct immune cell cluster (Kozlovski et al., 2025, DOI: https://doi.org/10.1101/2025.01.24.63469, preprint), in which *NveDicer1* and *NveAGO2* are enriched (Supplementary Figure 8). These results suggest that the siRNA pathway activation described here is likely mediated by immune cells and contributes to the antiviral response.

In response to this and related comments, we have revised the text. We have provided an explanation in the Introduction clarifying the absence of viral infection in our experimental design. We have included in the Discussion a disclaimer describing system limitations and emphasizing that our dsRNA and self-replicating RNA approaches simulate viral replication. We have expanded the discussion of our findings to note that the dsRNA-driven siRNA pathway likely serves an antiviral role in *Nematostella*, as supported by both previous studies and our current data

8. Line 58, the first report to document reverse transcription of viral material as part of antiviral RNAi in insects is Goic et al. (DOI: 10.1038/ncomms12410). Please add the citation.

Answer:

The citation has been added.

Reviewer #2: The paper from Admoni et al aims at understanding whether the siRNA pathway is functional in Nematostella vectensis and explores the biogenesis of exogenous small RNAs in this organism.
The experiments are correctly designed; however, some drawbacks are identified:

1) The results obtained don't support the statement that this study "characterizes the antiviral RNAi response" of Nematostella vectensis (L. 31, L. 249, L. 259, L. 273, L. 280, L. 308). The authors seem to confuse small interfering RNAs produced in response to viral infection with those produced after exogenous dsRNA injection. This study demonstrates that siRNAs are produced in Nematostella vectensis after injection of

exogenous dsRNA, while the production of siRNAs as a consequence of viral dsRNA synthesis during viral replication remains to be shown. So, although the manuscript provides support for the existence of an active siRNA pathway, further demonstration that this mechanism produces viral siRNAS, and reduces viral infection or replication would be the only way to reach the conclusion that the siRNA pathway is antiviral in Nematostella.

Answer:

Please refer to the response to reviewer #1 comment 7.

2) It is difficult to understand why the authors decided to silence GFP in transgenic animals. GFP is a stable protein, therefore the silencing/destruction of mRNA might not induce a sufficient or detectable decrease in protein amounts. The silencing of an endogenous gene with a clear phenotype (i.e. lethal or affecting the development) would have been a better choice or could be tested to complement these results and maybe see a stronger silencing effect.

Answer:

We would like to address the reviewer's concern by noting that GFP is frequently used in all types of knockdown assays and has also been used in *Nematostella* where its expression was significantly decreased by short-hairpin RNA (shRNA) delivery (Karabulut et al., 2019, DOI: https://doi.org/10.1016/j.ydbio.2019.01.005). Therefore, we believe that using GFP to assess silencing is not a limitation of the study.

3) Since I am not familiar with the animal model, it is not clear why experiments are done in embryos and not in adults. From the manuscript, it is not clear whether individual embryos were injected, and which RNA quantity was used (i.e. ng or pg per embryo).

Answer:

Currently, there is no established assay to deliver genetic material into *Nematostella* adults, and there is no available cell culture for *Nematostella*. Hence, microinjection into *Nematostella* zygotes is a commonly used approach to perform genetic manipulation. Using the typical microinjection setup, the volume of injection mix containing RNA/DNA molecules combined with a fluorescent tracer that is injected into each zygote is estimated visually. According to Layden et al., 2013 (DOI: 10.1038/nprot.2013.009), this volume is estimated as ~10 pl. Based on this estimation, the quantity of RNA that was injected into each individual *Nematostella* zygote in our eGFP dsRNA experiments is between 1.75e-05 to 7e-05 ng per embryo, depending on the concentration of the

injection mix. We have added this information to the manuscript in the Method section under Microinjection of dsRNA.

We would like to note that this quantity could be variable depending on the size of the microinjection needle, as it has to be adjusted manually and might change throughout the injection session (which generates one biological replicate) due to clogging of the needle.

4) It is not clear whether the different techniques for small RNA sequencing (standard, RppH treated, PANDORA-seq) were performed on the same RNA samples. This would perhaps explain the significant reduction in read counts in PANDORA-seq results compared to traditional sRNA sequencing (Figure 4C vs 4D)? Moreover, the authors should explain better what type of RNA modifications they expected to observe in small RNAs and how the PANDORA-seq technique removes these modifications.

Answer:

We have added a clarification that the same RNA was used to generate the small RNA libraries presented in Figure 2 and for the RppH treatment presented in Figure 3. A different set of biological replicates was injected for PANDORA-seq but the extracted RNA was used for both PANDORA and Traditional sequencing. We have added a clarification to the text.

We have added to the text the types of modifications PADNORA-seq removes. PANDPRA-seq method that was developed in the lab of Dr. Qi Chen (University of Utah) and uses a combination of two enzymes (T4 polynucleotide kinase and α-ketoglutarate-dependent hydroxylase) to remove these modifications. This information is available in the main text and in the Methods section in more detail. The PANDORA-seq method is published in the manuscript by Shi et al. 2021 (DOI: 10.1038/s41556-021-00652-7), where it is described in depth.

5) The Western blots in Figure 4E-I don't seem to add relevant information to the paper, as the regulation of RNAi proteins might be independent from the presence of dsRNA. For instance, in many organisms, viral infection or injection of dsRNA does not affect the transcription or the protein amounts of RNAi components. The use of a mimic of dsRNA (poly I:C) further weakens these results. Moreover, the Western blots are very confusing as the quantification in the plots (Figure 4I) are shown in opposite order from the samples at the left. Uncropped images of gels should be provided.

Answer:

In our previous publication (Lewandowska et al., 2021, DOI: 10.1093/molbev/msab197), we reported a strong upregulation (at the RNA level) of immune-related genes, including

AGO2 and *Dicer1*, in response to injection of poly(I:C) or eGFP dsRNA into *Nematostella* zygotes. Notably, poly(I:C) injection to *Nematostella* phenocopies the effects of dsRNA, inducing a similar transcriptional profile of immune-related genes (Figure 2 and Figure S5 in Lewandowska et al., 2021), Therefore, the use of poly(I:C) in the Western blot analyses presented here does not weaken our conclusions, as both molecules trigger a comparable antiviral response.

Consistent with these earlier findings, the present study demonstrates a strong upregulation of *AGO2* and *Dicer1* following injection of mRNA encoding Flock House Virus RdRp (Figure 4E-F). This shows that *Nematostella* might respond differently to the presence of dsRNA compared to other organisms, by upregulating RNAi components. Furthermore, Lewandowska et al., (2021) showed that knockdown of the dsRNA receptor RLRb abolishes the upregulation of *AGO2* and *Dicer1*, reinforcing the link between dsRNA sensing and induction of the siRNA pathway.

The Western blot results presented in this study further confirm that poly(I:C) exposure increases AGO2 and Dicer1 protein levels. In contrast, AGO1 and GW182, components specific to the miRNA pathway, show mild downregulation despite the shared machinery between the miRNA and siRNA systems. Together, these results indicate that dsRNA specifically activates the siRNA pathway while attenuating miRNA pathway activity, suggesting that Nematostella prioritizes siRNA-mediated responses as a preferred strategy against invasive dsRNA.

We thank the reviewer for noticing the confusing Figure. We have made it clearer and provided the uncropped Western images.

6) At L. 274 the authors say that the silencing of GFP is not long-lasting because of the absence of amplification via secondary production of siRNAs, but they didn't check if dsRNA was still present at later time points (i.e 48h or 72h) or if it was destroyed/degraded.

Answer:

We thank the reviewer for this helpful comment. To address it, we injected eGFP dsRNA to WT zygotes at three biological replicates and extracted RNA at 24 and 72 hours from the time of injection. We performed PCR using cDNA and eGFP-specific primers and endogenous gene primers as a positive control. The results show amplification of eGFP in both 24 and 72 hours timepoints, confirming that the dsRNA is stable in the injected animals within this time frame (Supplementary Figure 6C and D).

Minor comments:

- For transparency, the single point of each replicate should be visible in figures 2A, 2B, 3A, 3B, 4C, 4D.

Answer:

We thank the reviewer for this helpful suggestion. We have implemented it where possible by adding replicate points to Figures 2A, 3A, 4C, and 4D. However, adding replicate points to Figures 2B and 3B substantially increased visual noise and reduced clarity; therefore, we retained the original presentation for these panels to ensure the data remain clearly interpretable.

- Figure 3A: the use of the word "mock" here is confusing, as we might think these are sample that were not injected with dsRNA. Consider to change it to "no enzyme".

We agree with the reviewer's comment. We changed "mock" to "no enzyme" when describing of the reaction with no RppH treatment.

- Figure 3B: the color legend is incomplete (the RppH indication is missing).

Answer:

We corrected the color legend.

- Supplementary Figures start from #2

Answer:

We have corrected this error.

- Supplementary Files need an explanation/legend, especially #2

Answer:

We have added an explanation of the content for the supplementary files.

- Line 186: the number of the supplementary figure is missing.

Answer:

We have corrected this error.

- L. 329: at which concentration dextran was used? Was this quantity or concentration constant with varying dsRNA quantities? Did the author check for toxicity of this dye in their system to confirm that the mortality was linked to dsRNA injection rather than the dye itself?

Answer:

We have added to the concentration at which dextran Alexa Fluor 594 was injected (50 ng/µl). This concentration was constant across all injections in this paper (as mentioned in the Methods section under Microinjection of dsRNA and under FHV-eGFP and mCherry mRNA generation and injection). As animals injected with mock mix, without dsRNA (Figure 2A), showed higher survival, the presence of dextran was not the cause of the high mortality of the dsRNA-injected animals. In addition, dextran is broadly used as a fluorescent tracer in microinjections of *Nematostella* (and other cnidarians) and is not known to have biological effects (Layden et al., 2013, DOI:10.1038/nprot.2013.009, Paix et al. 2022, DOI: https://doi.org/10.1073/pnas.2215958120).

- L. 366: please indicate cDNA quantity rather than volume.

Answer:

We changed the indication to cDNA quantity.

- L. 393: a specific paragraph explaining bioinformatic analyses should be created.

Answer:

We have created a separate paragraph for bioinformatic analysis.

- The term RNAi should be only used to describe the global mechanism encompassing 3 pathways: siRNA pathway, miRNA pathway, and piRNA pathway.

Answer:

We agree with the reviewer's comment. We have corrected the text accordingly.

Reviewer #3: In this manuscript, Admoni et al. investigate RNA interference in the model cnidarian Nematostella vectensis. Authors used dsRNA injection to induce RNAi and analyzed its efficiency, small RNA production, and expression of genes associated with RNAi and miRNA pathways. Authors conclude that Nematostella is possessing a simple RNAi pathway without secondary piRNA production and propose that RNAi in this model species might function only as a short term antiviral mechanism.
Overall, this work is in line with other studies testing RNAi activity by dsRNA injection. What makes this work relevant and of interest of a broader readership is that it is using an experimental model from early branching metazoa. While RNAi in Cnidaria was demonstrated already in 1999 (https://doi.org/10.1006/dbio.1999.9407), experimental

analysis of the molecular mechanism presented here provides a valuable example of adaptation of animal RNA silencing pathways and dsRNA response in a lineage, which separated from animals before the lineages of the main laboratory model organisms branched. At the same time, I think the work requires additional data analyses and more critical interpretation of data because authors tested just dsRNA response rather than innate immunity/antiviral response.

Major

1) As indicated above, I do not think authors truly reveal antiviral RNAi in Nematostella as the work is addressing it indirectly. Therefore, this work characterizes canonical RNAi in Nematostella, which has potential to have antiviral role. I ask authors to avoid the term antiviral RNAi in the results section and discuss (obvious but not tested) implications for antiviral immunity in Nematostella in the discussion.

Answer:

Please refer to the response to reviewer #1 comment 7.

2) While authors observed modest RNAi, the profile of small RNAs mapped onto the EGFP sequence suggests that injected dsRNA is efficiently converted to dsRNA. It is thus possible that poor efficiency of RNAi is caused by poor loading of Dicer-generated small RNAs into the AGO2 protein. Authors should either 1) point out that their RNA-seq analysis cannot distinguish from AGO2-bound and unbound fraction and thus it is unclear how limiting is AGO2 loading for RNAi efficiency or 2) employ the TraPR approach(https://doi.org/10.1093/nar/gkaa472) to isolate and determine the fraction of AGO2-bound small RNAs. The mapping reoccurring pattern suggest it may be the majority

Answer:

We thank the reviewer for this valuable suggestion. We have added two experiments to the manuscript, in which we applied two different methods of AGO isolation in order to capture AGO-bound eGFP-mapping siRNAs. The first was immunoprecipitation of AGO2 using verified AGO2 antibodies and the second was Trans-kingdom rapid affordable Purification of RISCs (TraPR), as the reviewer suggested. We injected WT animals with eGFP dsRNA and collected them at 24 hpi. Following AGO isolation we performed small RNA sequencing, which for both methods shows that eGFP-mapped reads at the size of 20-21 nucleotides are highly enriched in the AGO-isolated samples (Supplementary Figure 4A-D). This validates the loading of eGFP dsRNA-derived siRNAs into the RISC complex. To assess the loading efficiency, we compared the total read counts of dsRNA-derived siRNA to the counts of miRNAs captured by the two

methods and showed that they are significantly higher (Supplementary Figure 4E and F). Since miRNAs are typically loaded efficiently into AGO and their levels are not affected by dsRNA treatment (Supplementary Figure 7), this comparison demonstrates that dsRNA-derived siRNAs are loaded into AGO2 with high efficiency.

3) I think characterization and discussion of small RNA patterns is confusing if not misleading for a non-expert reader and should be revised. First, it should be clearly stated whether the dsRNA structure allows for processing from ends. Then, authors should use a phase analysis (DOI: 10.1126/science.1241930) to examine whether this dsRNA is predominantly processed from ends or from stochastically initiated internal cleavage. It is important to point out that the reoccurring pattern likely reflects strand selection and AGO2-loaded siRNAs but it should not be compared with patterns observed by Flemr et al. 2013 (10.1016/j.cell.2013.10.001) who used long dsRNA hairpin with inaccessible ends. If authors used long dsRNA with accessible ends, the pattern in Fig. 2 likely reflects efficient processive cleavage (no accumulation of signal at dsRNA termini (but possibly positive phasing signal) and loading (the pattern is not symmetrical).

Answer:

We thank the reviewer for this insightful comment. The dsRNA used in our experiments was generated by annealing complementary single strands and therefore lacks a loop structure, allowing processing from both ends. We have added this clarification to the manuscript (Figure 1A and text). In addition, we now include a phasing analysis and discuss its implications for Dicer processing (see response to reviewer #1 comment 4). We agree with the reviewer that the recurring mapping pattern likely reflects efficient processive cleavage, which is consistent with the weak phasing observed. Our comparison to Flemr et al. (2013) specifically refers to the reproducible mapping pattern seen across experiments (Figure 7B in Flemr et al.), suggesting that Dicer exhibits sequence preferences at processing sites. Similarly, in our data, comparable siRNA patterns were detected across independent replicates, supporting this interpretation.

Minor issues

1) Line 25. The RNAi definition sounds odd and partial - it is missing the sequence-specific mRNA degradation guided by siRNA. The original definition stemming from Fire et al., 1998 was sequence-specific mRNA degradation induced by long dsRNA. Later it would integrate that degradation is guided by small RNAs produced from long dsRNA.

Answer:

We have edited the text to include the original definition.

2) I think using RNAi in the introduction as a common term for RNA silencing or small RNA pathways is confusing despite some authors are using RNA interference as a common term for RNA silencing pathways.

Answer:

To address this comment, as well as the comment by reviewer #2 (using the term RNAi only to describe the global mechanism encompassing three pathways), we have edited the Introduction to include a more concise definition of RNAi.

3) Fig. 1 - it would be reassuring if authors would include some quantitative analysis of EGFP signal.

Answer:

We thank the reviewer for this comment. We have added a quantification of fluorescence intensity to the manuscript (see response to reviewer #1 comment 1).

4) Please, include a scheme of the transgene & dsRNAs, ideally drawn to a scale in Fig.1.

Answer:

We have added a scheme of the transgene and dsRNA as the reviewer requested (Figure 1A).

5) Fig. 1 - panels C and D should be merged into one graph for easier comparison.

Answer:

The panels have been merged the reviewer requested.

6) I'm sorry, I do not understand the read normalization and Y scale in sequencing data. Does the panel Y in Fig. 2A imply that the scale is in hundreds of millions of normalized reads (10^5 on the axis x 10^5 from the Y axis title). I would very much prefer to have the value expressed as the number of reads per million of mapped 18-30 nt reads in the library as it gives much better perception of the small RNA abundance and allows for a simple comparison with published data and available dasatets.

Answer:

We thank the reviewer for this comment. We have updated the Y-axis of all small RNA sequencing graphs to display read counts normalized to spikeins. In addition, we

calculated total read counts for our main small RNA sequencing datasets in Reads Per Million (RPM) (Supplementary Figure 1). Whenever possible, we retained spikeins normalization in the main figures (excluding AGO2 immunoprecipitation, TraPR and PANDORA libraries, where read counts are shown as RPM), because spikeins normalization provides a more accurate and quantitative comparison of small RNA abundance across samples.

7) Were any EGFP-derived piRNA-like small RNAs observed? Please, comment.

Answer:

Based on the size distribution of eGFP mapping reads (Figure 2B, 3B, G, and H) that show peaks at 20-21 nucleotides, we believe that it is unlikely that any eGFP-derived piRNAs can be observed in our data, as piRNAs are characteristically longer (Matranga and Zamore, 2007, DOI: 10.1016/j.cub.2007.07.014).

8) p13, l 327 - please, describe the dsRNA in detail here. This includes the length (needed to estimate molarity of injected dsRNA), and form (blunt ends, some overhangs, dephosphorylated or with triphosphates from in vitro transcription, just annealed or purified to remove ssRNA … etc.).

Answer:

We have added the information to the manuscript. The dsRNA carrying eGFP sequence was 720 bp for full-length and 360 bp for half-length. It included 5′ triphosphate and blunt ends and was prepared by the companies by annealing of single strands followed by column purification.

9) p13, l 332 - please, include the microinjected volume and add somewhere (e.g. into line 324) the diameter (or its range) of the microinjected zygotes. This information is necessary to have an insight into the stoichiometry of silencing and siRNA production.

Answer:

We added the estimated volume of microinjection and the diameter of *Nematostella* eggs into the Methods section under Microinjection of dsRNA.

10) Discuss the stoichiometry and quantity of dsRNA and siRNA in Nematostella zygote and compare that with known data from mice. In the initial experiment (10.1242/dev.127.19.4147), authors injected ~1 000 000 and 10 000 dsRNA molecules into mouse oocytes with a diameter of 80 micrometer. Could authors bring up and discuss parameters of their experimental model (number of microinjected dsRNA

molecules, their length, possible small RNA molarity if all dsRNA would be converted to siRNA … this framework is important to understand whether Nematostella has simple but relatively efficient RNAi standing on processive dsRNA cleavage or inefficient RNAi comparable to that observed in mammals.

Answer:

We would like to refer to our response to reviewer #2 comment 3. We note that due to technical constraints of microinjection to *Nematostella* zygotes, we can only roughly estimate the volume injected into each zygote; hence the estimated number of dsRNA molecules injected could also be inaccurate. Based on the estimated volume of injection (~10 pl), the number of injected molecules is between 2.22e+04 to 8.87e+04 (of 720 bp dsRNA). The diameter of *Nematostella* eggs is 200–300 μm (Fritzenwanker et al., 2007, doi: 10.1016/j.ydbio.2007.07.029). If all dsRNA is converted into siRNAs, theoretically the concentration of siRNAs within an egg would be ~0.3–1.2 nM, but it is important to remember that quickly after injection the eggs divide, and the effect is quantified at 24 hours post-injection. Finally, we think that comparison of our experimental setup in *Nematostella* to experiments performed in mouse oocytes might lead to inaccurate conclusions, due to the difference between the eggs (*Nematostella* eggs do not contain yolk), the experimental setups, and the variability and rough estimation of the volume injected.

11) The work should include a reference to Grimson et al. (10.1038/nature07415) 2008 and clarify the number of AGO proteins - they state that Nematostella has 2 Dicers and 3 AGOs.

Answer:

In the study by Moran et al. (2013; DOI:10.1093/molbev/mst159), the authors report, in reference to Grimson et al., that contrary to the earlier claim that *Nematostella* possesses three AGO genes, it actually has only two. The putative third *Nematostella* AGO gene was identified as an artifact of genome assembly, as its predicted model corresponds to a short fragment nearly identical in nucleotide sequence to a portion of *Nematostella AGO2*. We added a citation of Moran et al. (2013) in the manuscript.

12) AGO1 and GW182 depletion in the presented western blot is rather mild. These data should be approached with caution as densitometry has typically unknown linearity range and, in the absence of serial dilution of the sample, even a 2-fold change in the protein abundance may be quite difficult to estimate.

Answer:

We thank the reviewer for this comment. We have revised the text to describe the downregulation of miRNA pathway components as “mild.” We would like to emphasize that the key finding of this experiment is not the precise magnitude of the downregulation, but rather that siRNA pathway components are strongly and specifically upregulated in response to poly(I:C) injection, whereas miRNA components are not.
